# Genetic mapping and quantitative trait loci analysis for pistillate flowers per node and multi-pistillate flower traits in the F_2_ cucumber population

**DOI:** 10.1270/jsbbs.23070

**Published:** 2024-06-18

**Authors:** Nattawat Anankul, Wannapa Sattayachiti, Namfon Onmanee, Saengchit Chanmoe, Weenun Bundithya, Jutamas Kumchai

**Affiliations:** 1 Department of Plant and Soil Sciences, Faculty of Agriculture, Chiang Mai University, Chiang Mai 50200, Thailand; 2 Hortigenetics Research (S.E. Asia) Company Limited, Chiang Mai, 50290, Thailand

**Keywords:** cucumber, multi-pistillate flower, QTL, markers, breeding

## Abstract

This study focused on cucumbers’ multi-pistillate flower (MPF) trait, which is essential for high yields. A genetic linkage map was constructed using a population of 219 F_2_ plants to analyze quantitative trait loci (QTL) associated with MPF traits. Crossbreeding of EWSCU-809 (MPF) with EWSCU-989 (single pistillate flower: SPF) generated an F_1_ hybrid that self-pollinated to form an F_2_ population. Based on 244 single nucleotide polymorphic markers across seven cucumber chromosomes, a linkage map facilitated QTL analysis considering average pistillate flowers (PFs) per node and nodes with MPF traits. The results indicated a 9:6:1 epistatic ratio in the F_2_ populations, revealing recessive allele control of the MPF trait in gynoecious plants. Three QTLs (*qMP2.1*, *qMP3*, *qMP7*) on chromosomes 2, 3, and 7 were associated with average PFs per node, explaining 5.6 to 10.3% of phenotypic variation. Four QTLs (*qMP2.2*, *qMP3*, *qMP4*, *qMP7*) on chromosomes 2, 3, 4, and 7 were linked to the presence of nodes with MPF traits, explaining 5.8 to 10.6% of phenotypic variance. Notably, QTL regions overlapped between the two datasets, suggesting pleiotropic effects, particularly on chromosomes 3 and 7. These reliable QTLs have the potential to improve breeding programs, enhance PF development, and increase cucumber yields.

## Introduction

**Fig. 1.**
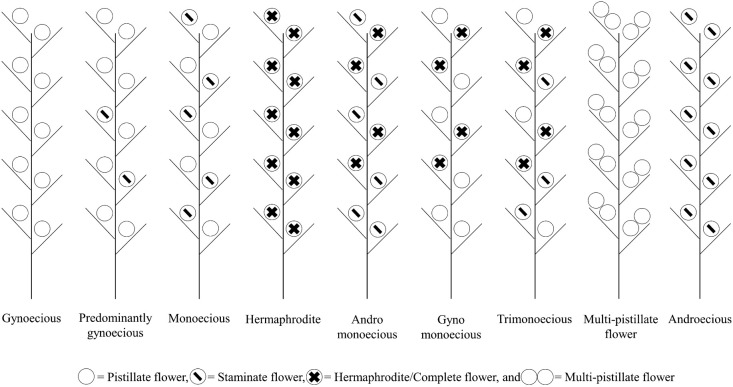
Sex expression types in cucumber (figure modified from Li *et al.* 2019).

The cucumber (*Cucumis sativus* L.) belongs to the Cucurbitaceae family and is an economically important vegetable crop worldwide (Simi *et al.* 2017). While vegetables account for 12% and represent the top three in world crop production by commodity group, in the vegetable group, cucumber has an 8% global share, following tomatoes (16%) and dry onions (9%) (FAO 2021). The cucumber vine displays three main flower types: staminate (male), pistillate (female), and hermaphrodite (bisexual/perfect), and two minor genes with the *M* gene that regulate unisexual flowers. Numerous studies on cucumber research have identified three genes, *F* (femaleness), *m* (andromonoecy), and *a* (androecy), that control sex determination in cucumbers. These genes are linked to the 1-aminocyclopropane-1-carboxylate synthase (*ACS*), a crucial regulatory enzyme in the ethylene biosynthetic pathway. Genes involved in ethylene signaling pathways may influence the unique development of sex organs (Li *et al.* 2009, Matsumura *et al.* 2014, Mibus and Tatlioglu 2004, Shyam *et al.* 2020). The most prevalent cucumber plant is the unisexual line, specifically monoecious (*MMffAA*), which exhibits female (pistillate) and male (staminate) flowers on the same plant. The second type is gynoecious (GY: *MMFFA/a*), which exclusively produces pistillate flowers; each node may have one or more female flowers. This trait may not be ideal in specific production systems with suboptimal cultural practices or poor production conditions because not all female flowers develop marketable fruits. The third type is predominantly gynoecious (PGY: *MMFfA/a*), wherein the plant displays pistillate and staminate flowers (Bu *et al.* 2016, Li *et al.* 2009, Verma *et al.* 2018). The other unisexual line is androecious (*MMffaa/mmffaa*), with only staminate flowers on individual plants. Cucumber plants (bisexual lines) exhibit other sex expression types, including those that produce hermaphrodite (*mmFFA/a*) flowers containing both staminate and female parts on the same flower. The following common types are andromonoecious (*mmffAA*), gynomonoecious (*gym*), and trimonoecious (*tr*) bearing staminate and hermaphrodite, pistillate and hermaphrodite, and pistillate, staminate, and hermaphrodite flowers on the same plant, respectively (Fig. 1) (Li *et al.* 2009, Megharaj *et al.* 2017). Many factors affect sex expression, such as plant growth regulators (Adhikari *et al.* 2012), light induction based on season and day length, for example, blue light that favors pistillate flower formation (Zhou *et al.* 2018), and all the factors affecting gene control of *F* (Mibus and Tatlioglu 2004, Pawełkowicz *et al.* 2019), *m*, a, and *tr* (Kubicki 1969, Li *et al.* 2009).

Cucumbers generally produce SPFs or a single fruit per node. Double and plural pistillate flowering are also called multi-pistillate flowering (MPF). The identification of a single gene initially referred to as *mp* by Nandgaonkar and Baker (1981), has provided insights into the genetic foundation of MPF per node in specific plants. Subsequent research by Fujieda *et al.* (1982) suggests a correlation between *mp* and its designated gene *pf*, which has three distinct alleles: *pf^+^* for a single pistillate, *pf^d^* for a double pistillate, and *pf^m^* for MPF. Remarkably, the incomplete dominance exhibited by these alleles introduces an additional layer of complexity, underscoring the intricate genetic mechanisms governing pistillate flowering variations. This collaborative effort by various research groups provides valuable insights into the genetic regulation of flower development, paving the way for further exploration and comprehension of plant genetics. MPF, which corresponds to the bearing of two or more cucumber flowers/fruits per node, can save some of the work involved in pruning and vine training and provides the advantage of producing a large number of fruits (Fujieda *et al.* 1982). Yuan *et al.* (2008) and Dou *et al.* (2010) reported that the number of fruits per plant is closely related to cucumber yield and has major potential for increasing this. Moreover, the PF ratio influences the fruit per plant or yield.

Plant biotechnology is a valuable and practical plant breeding tool. Many techniques have helped breeders identify or confirm genetic control in hybrids, interspecific hybrids, and other trials (Kumchai *et al.* 2013). Marker-assisted selection (MAS) is an advanced molecular breeding program that supports conventional breeding (Das *et al.* 2017). Genotyping technology has enabled the cost-effective development of single nucleotide polymorphism (SNP) markers. Moreover, as reported in many crop breeding schemes, the MAS method can be used for various purposes, such as marker-assisted backcrossing (Lee *et al.* 2022). It can also help identify the Rf/rf locus and normal/sterile cytoplasm in chili breeding programs (Na Jinda *et al.* 2023). Improving cucumber traits is essential for breeding programs (Yuan *et al.* 2008). In this regard, the environment has a strong effect on phenotyping; for example, the sex expression of cucumber at high temperatures and long photoperiods results in cucumbers having less PF (Fujieda *et al.* 1982, Megharaj *et al.* 2017). Therefore, molecular markers may increase the efficiency and accelerate plant breeding programs. Moreover, due to environmental effects, MAS may help select desirable traits that are not present in the field (Collard *et al.* 2005). SNPs are the basis for differences between alleles and can be used as simple genetic markers to identify the position of virtually every gene. They have great potential for detecting associations between the allelic forms of genes and phenotypes. Analysis of SNP haplotypes provides an effective way to associate alleles with traits (Rafalski 2002). SNP markers can distinguish between homozygous and heterozygous phenotypes, allowing many samples to be rapidly identified and genotyped (Junta *et al.* 2020). Therefore, this study aimed to pinpoint SNP markers linked to MPF traits by utilizing the F_2_ population resulting from a cross between female MPF and male SPF traits. The primary objective was to identify stable varieties through genotyping and molecular marker techniques in horticultural genetics, providing a guideline for supporting the improvement and development of cucumber varieties alongside conventional methods.

## Materials and Methods

### Plant materials

The plant materials comprised a wide range of cucumber varieties. Parental lines comprising four MPF and two SPF varieties were obtained from Hortigenetics Research (S.E. Asia) Ltd.

### Evaluation and homogeneity testing for parental line selection in cucumber varieties

**Fig. 2.**
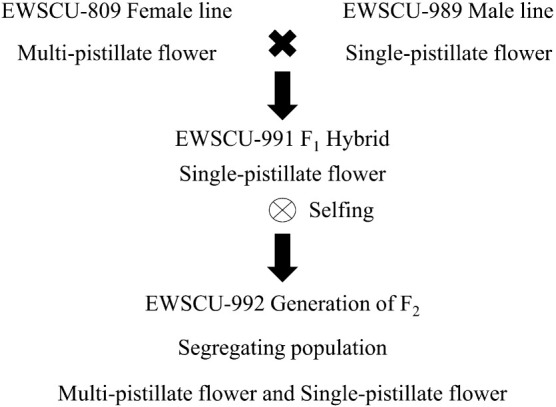
The crossing between parental lines to produce F_1_ and F_2_ populations for QTL mapping and analysis.

Two to three young leaves from the upper portion of the cucumber plants were sampled for homogeneity testing. We employed a set of 70 SNP molecular markers derived from the transcriptome resequencing of cucumber varieties from Hortigenetics Research (S.E. Asia) Ltd., utilizing the KASPar technique. This dataset served as the foundation for selecting parent varieties to establish F_1_ and F_2_ population databases. The F_1_ hybrid was the result of crossing an inbred EWSCU-809 (MPF) line as the female parent with EWSCU-989 (SPF) as the male parent. Subsequently, the F_1_ generation underwent self-pollination, yielding 219 plants in the F_2_ population (Fig. 2), which were sown under greenhouse conditions during the rainy season of 2021, and individual plants were singled out for branch analysis. Data collection for the F_1_ and F_2_ parental lines involved observing pistillate flower numbers on the main stems from the 5^th^ to 25^th^ nodes. Individual plant data included the average number of pistillate flowers per node and the number of nodes with MPF traits. This information facilitated the calculation of heterosis and the identification of dominant and recessive traits.

Evaluation and homogeneity testing were conducted to select parental lines of the cucumber varieties. Two to three young leaves from the cucumber plant upper portions were sampled for homogeneity testing. A set of 70 SNP molecular markers derived from the transcriptome resequencing of cucumber varieties from Hortigenetics Research (S.E. Asia) Ltd., was employed using the KASPar technique. This dataset formed the basis for selecting parent varieties to establish the F_1_ and F_2_ population databases.

The F_1_ hybrid was obtained by crossing an inbred line with EWSCU-809 (MPF) as the female parent and EWSCU-989 (SPF) as the male parent. Subsequently, the F_1_ generation was self-pollinated, yielding 219 plants in the F_2_ population (Fig. 2). These plants were sown under greenhouse conditions during the rainy season of 2021, and individual plants were selected for branch analysis. Data collection for the parental lines F_1_ and F_2_ involved observing PF numbers on the main stems from the 5^th^ to 25^th^ nodes. Individual plant data included the average PF numbers per node and the number of nodes with MPF traits. This information facilitated the calculation of heterosis and the identification of dominant and recessive traits.

### DNA extraction and Polymerase Chain Reaction (PCR)

Young true leaves on the main stem of parental lines F_1_ and F_2_ were collected for DNA extraction using the cetyltrimethylammonium bromide (CTAB) method, and genomic DNA was diluted to a final concentration of 15 ng/μL as described by Nishiguchi *et al.* (2002).

PCR primers forward 1 (F1), forward 2 (F2), and reverse (R) were used for the PCR. A buffer of KASPar master mix (2X) and primers were added to the assay plate. Next, a DNA 15 ng/μL template was prepared and put in a DNA plate. The assay and DNA plate were centrifuged for 20 min at 3,500 rpm (1178 × g) and placed into a Nexar machine for subsequent dispensing onto array tape^TM^ (384 holes). The DNA and assay array tape was then sealed with a plastic cover tape.

Afterward, the array tape was placed in a Thermal Cycler (Soellex) to increase the DNA and centrifuged for 10 min at 5,000 rpm (2404 × g) (total 2 hours). The results were analyzed using Nexar-Araya system Intellics^®^ software v 1.0.0.0. All processes used ultra-high-throughput SNP technology (Douglas Scientific).

### Primer screening for polymorphic markers and identification and substitution mapping of quantitative trait loci (QTL) for MPFs

A total of 1,914 SNP markers were designed to cover all seven cucumber chromosomes and screened to identify polymorphism in female, male, and F_1_ hybrids. SNP genotyping was performed using the KASPar^TM^ technology by KBioscience^®^. In the two fluorescence resonance energy transfer (FRET) cassettes, a primer was conjugated to a fluorescent dye (VIC, FAM, or heterozygous) (Raitio *et al.* 2012), and the DNA was amplified using specific primers. When the FRET cassette primer hybridized to DNA, the quencher was separated (Saeko *et al.* 2020).

The average number of PFs per node and the number of nodes with MPF traits of individual plants from 219 F_2_ plant populations were used for QTL mapping, resembling linkage analysis with the JoinMap4.0 program and MapQTL 6 software. Out of the 1,914 SNPs screened, 244 were found to be polymorphic. Marker data were calculated using the logarithm of odds (LOD) with a multiple QTL model.

## Results

### Evaluation and homogeneity testing for parental line selection of cucumber varieties

**Fig. 3.**
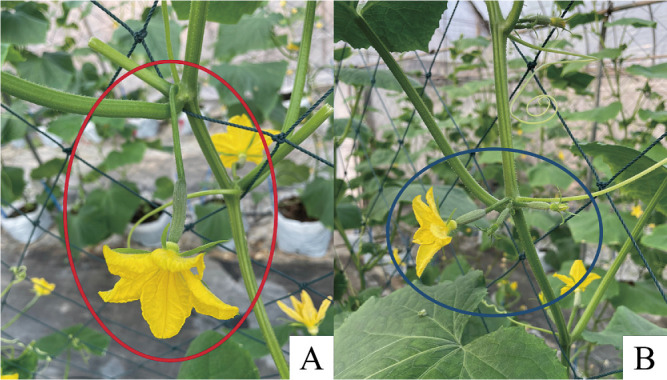
Single-pistillate flowers shown by the red circle (A), multi-pistillate flowers shown by the dark blue circle (B).

EWSCU-809 and EWSCU-989 were chosen as the female and male lines, respectively, demonstrating a GY-type with MPF and PGY with SPF. Furthermore, they exhibited impressive homogeneity, with 97.14% in the female line and 95.24% in the male line (Table 1, Fig. 3).

### MPF traits in parental lines, F_1_, and F_2_ populations

**Fig. 4.**
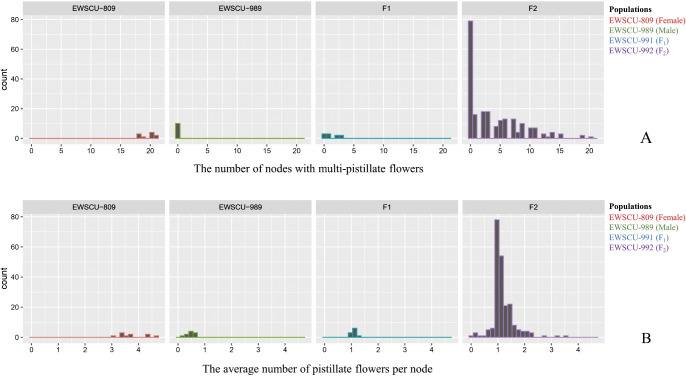
The frequency distribution histograms of four populations (10 plants of the female and male lines, F_1_ hybrid, and 219 F_2_ populations). The average number of pistillate flowers per node (A) and the number of nodes with multi-pistillate flowers (B).

P1 exhibited the highest number of female flowers per node (3.76) and the highest number of nodes with MPF traits (19.50), results markedly different from those of P2 and F_1_. Heterosis yielded negative results in both sets of data. Within the F_2_ population, there was a segregation in the flowering phenotype, distinguishing between MPF and SPF traits and variations in the number of female flowers per node and the number of nodes with MPF traits (Table 2, Fig. 4).

The distribution and chi-square (χ^2^) estimates of F_2_ populations comprising 219 plants were analyzed for the average number of MPFs per node and the presence of MPF traits in nodes. The populations were categorized based on the ratio of non to partial to strong MPFs, and the observed ratio was 9:6:1 in both the two-parameter studies (Table 3).

### Markers screening

**Fig. 5.**
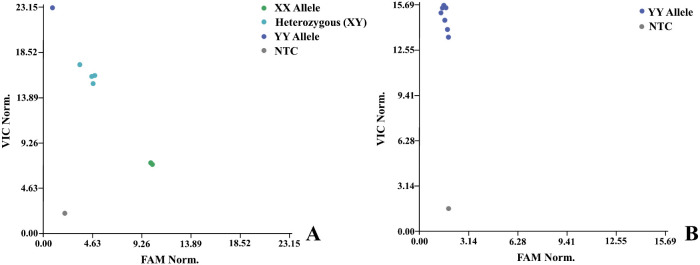
Polymorphic (A), and monomorphic (B) marker.

**Fig. 6.**
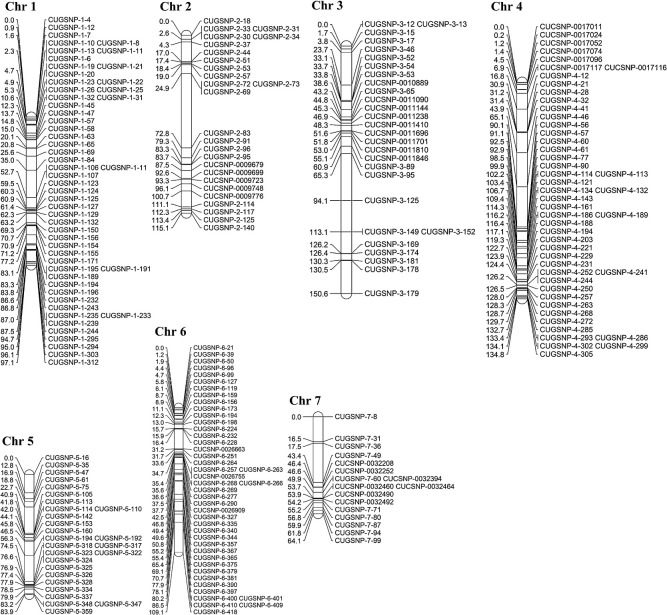
A physical map of 244 polymorphic markers on 7 chromosomes.

In total, 1,914 markers were screened for female and male lines and F_1_ hybrids. These were separated into polymorphic (Fig. 5A) and monomorphic (Fig. 5B) variants. Distributed across seven chromosomes, 244 polymorphic SNP markers (Fig. 6) were selected for subsequent QTL analysis.

**Fig. 7.**
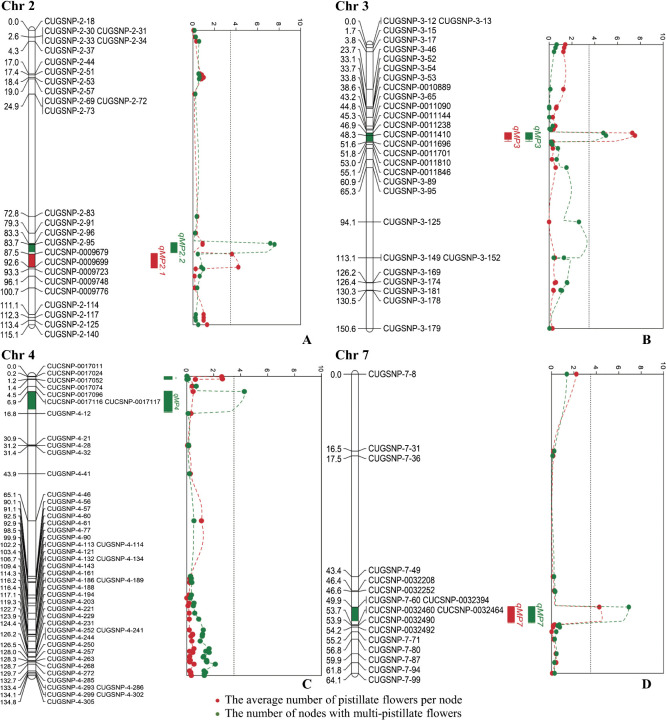
A physical mapping of 7 QTL positions, using the average number of pistillate flowers per node and the number of nodes with multi-pistillate flowers from 219 F_2_ population plants, detected on chromosome 2 (A), chromosome 3 (B), chromosome 7 (C), and chromosome 4 (D).

The QTL of the average number of MPFs per node and the number of nodes with MPF traits were analyzed using LOD scores. The results of the average number of MPFs per node revealed three notable QTLs on chromosomes 2, 3, and 7, which are qMP2.1, qMP3, and qMP7, with LOD scores of 4.23, 7.53, and 4.53, PVE values of 5.6, 10.3, and 5.9%, and DOM values of –0.068, –0.083, and –0.004, respectively. Based on the number of nodes with MPF traits, four important QTLs were detected on chromosomes 2, 3, 4, and 7, which are qMP2.2, qMP3, qMP4, and qMP7, with LOD scores of 7.58, 4.99, 4.29, and 6.9, PVE values of 10.6, 6.8, 5.8, and 9.6%, and DOM values of –0.101, –0.108, –0.526, and 0.118, respectively (Table 4, Fig. 7).

## Discussion

The female line, characterized by GY, is commonly employed as the female parent to create hybrid lines and for cucumber seed production. This choice was made to enhance fruit production per plant, benefiting farmers through increased yields and higher incomes. It is advantageous for cucumber plants to have MPFs per node and numerous nodes with abundant PFs (More 2001). This study focused on two parental lines exhibiting distinct phenotypes. The first was the MPF-type EWSCU-809 (female line: inbred line), characterized by 3.76 PFs per node. In contrast, the SPF-type EWSCU-989 (male line: inbred line) features 0.46 PFs per node. EWSCU-809 had 19.50 nodes with multiple PFs, whereas EWSCU-989 had one PF per node. Following crossbreeding, EWSCU (809 × 989: F_1_; EWSCU 991) manifested a heterozygous phenotype. The presence of one SPF (one flower per node) indicated that its genotype closely resembled that of a homozygous dominant male parental line. MPFs in the female line signify a homozygous recessive trait (Miko 2008a). The F_2_ population (EWSCU 992) exhibited pronounced segregation of SPFs and MPFs. Identifying a single gene initially referred to as *mp* by Nandgaonkar and Baker (1981), has provided insights into the genetic foundation of MPFs per node in specific plants.

The segregation pattern in the F_2_ generation adhered to Mendel’s law of segregation, with selfing of the F_1_ generation resulting in a segregation ratio of 1:2:1. There were two alleles in a gene pair, one of which was completely dominant, resulting in a 3:1 ratio. Mendel’s law explained the relationship between these phenotypes based on observations of both the F_1_ and F_2_ generations. This study revealed a 9:6:1 segregation ratio for both characteristics in the phenotype of the F_2_ generation, indicating the influence of epistatic interactions, which implies complete dominance of both gene pairs. However, when one gene was dominant, the other was suppressed. The epistatic ratio type comprises duplicate genes with cumulative effects or additive gene interactions (Huang *et al.* 2021, Miko 2008a). Hence, MPF traits in gynoecious cucumber lines are governed by a recessive allele, and genetic analysis suggests the involvement of one (*mp*) or two major genes and several modifying factors that influence this characteristic. In contrast, the dominant allele controls the SPF (Nandgaonkar and Baker 1981). The genotype determines the phenotype, with the environment contributing as another influencing factor (Miko 2008b). These results indicate that the F_2_ population serves as a fundamental and advanced model for studying Mendelian genetics, making it well-suited for genetic investigations in both conventional and molecular breeding. It illustrates key genetic concepts, such as dominance, epistasis, linkage, and statistical predictions (Miko 2008c).

Cucumber horticultural characteristics are important traits controlled by QTL. With the exponential increase in cucumber QTL mapping studies, the naming of quantitative traits and the corresponding genes and QTL responsible for key phenotypic traits (Sheng *et al.* 2019, Wang *et al.* 2020). Numerous traits associated with flowering time (FT) and sex expression in cucumber plants are correlated, suggesting potential regulation by common ethylene-related pathways, as exemplified by the identification of QTL clusters on chromosomes 1, 3, 5, 6, and 7 in cucumber (Wang *et al.* 2020). Here, examination of flowering types, male flowers per node (MPF), and nodes with MPFs in GY cucumber plants revealed consistent chromosome locations on 3 and 7, denoted as *qMP3* and *qMP7*, respectively, implying potential pleiotropic effects. Additionally, other cucumber traits exhibited similar pleiotropic effects, as evidenced by Wang *et al.* (2021), who identified a pleiotropic *B* locus on Chr4 controlling fruit ground color at maturity and fruit spine color in Sikkim cucumbers. Furthermore, studies by Somta *et al.* (2020) on black gram (*Vigna mungo* (L.) Hepper) confirmed the pleiotropic effect of the mog gene on linkage group 6. The study was observed across various traits, including seed length, seed width, leaf length, leaf width, leaf area, pod width, straight pod length, and stem plant height represented by *qSdl6.1* and *qSd100wt6.1*, *qSdwa6.1*, *qLfl6.1*, *qLfw6.1*, *qLfa6.1*, *qPdw6.1*, *qPdl6.1*, and *qPlh6.1*, respectively. Notably, QTL mapping of cucumber horticultural traits validated 36 QTLs associated with FT, fruit length (FL), fruit diameter (FD), fruit shape (LD), fruit number (FN), and powdery mildew resistance. Among these, five moderate or major effect QTLs for FL, FD, LD, and FN within the inversion likely indicate the pleiotropic effects of andromonoecy (*m*) or the *cn* locus (Pan *et al.* 2020). The findings presented in Table 4 indicate that LOD scores >3 signify the presence of a molecular marker at a position correlated with the QTL. The QTL of MPFs of chromosomes 3 exhibited a phenotypic variance of 10.3% (Table 4), indicating a marked genetic segregation in the F_2_ generation, particularly in the homozygous recessive genotype. Han *et al.* (2010) demonstrated that the five QTL of MPFs in cucumber progeny exhibited substantial phenotypic variance, surpassing 10% across six QTL cultivated during both the autumn and spring seasons. Klosinska *et al.* (2006) reported that a pair of PFs was recessive and remained unexpressed in the F_1_ hybrid generation. This study observed the absence of a major QTL, possibly because this trait is influenced by environmental factors such as day length, air temperature, and photoperiod. For example, elevated air temperatures may decrease the presence of PFs, whereas lower air temperatures may increase their occurrence (Hikosaka and Sugiyama 2004). Han *et al.* (2010) highlighted that QTL and LOD scores in cucumber recombinant inbred lines of the F_9_ generation varied between seasons, specifically between spring and autumn, and across different years. Sheng *et al.* (2019) indicated that QTL *FT6.3* contributed to photoperiod-sensitive flowering time during domestication, whereas QTL *FT1.1* played an important role in regulating flowering time in cultivated cucumbers. Sex expression in Cucurbitaceae is influenced by several factors (Li *et al.* 2019). Ethylene is a crucial element that induces PF in cucumber (Manzano *et al.* 2013). Consistent with prior research, Li *et al.* (2019) emphasized that ethylene, which functions as a plant hormone, plays a pivotal role in initiating PF formation and determining the PF ratio (Megharaj *et al.* 2017, Pan *et al.* 2018). Moreover, Dou *et al.* (2015) demonstrated that the ratio of pistillate to staminate flowers was season-dependent. Therefore, strategically selecting markers could contribute to developing cucumber varieties with stable yields and consistent sex expression. Considering these findings, the identified QTL could serve as valuable tools for MAS in breeding programs focused on MPF traits. Furthermore, QTL qMP3 was identified at the same genomic position on chromosome 3 and QTL qMP7 at the corresponding position on chromosome 7. This observation applies to data patterns involving the average number of PF per node and the incidence of MPF traits in the F_2_ population. Genetic and environmental factors influence sex expression in cucurbits. Therefore, the breeding program needs to consider and control flower sex for a successful program. The MPF per node is a recessive trait less pronounced in F_2_ populations. To further advance the study of MPF for cucumber improvement, an increased population of plant samples for thorough examination and additional screening of primers for polymorphic markers and identification is necessary for result validation. Moreover, investigating QTL related to PF on the cucumber chromosome is a focal point for studying cucumber breeding programs. Identifying high yields per plant through this observation could benefit plant breeders aiming to create new varieties by modifying conventional methods, such as backcross breeding and hybrid cross. Incorporating molecular techniques would expedite gene transfer to target plants, streamlining the overall process.

In summary, the investigation of the cucumber parental lines EWSCU-809 and EWSCU-989 revealed a notable level of homogeneity but displayed negative heterosis in the F_1_ generation. The F_2_ generation exhibited marked segregation in MPF traits and the number of nodes with MPFs. Identification of four QTL on chromosomes 2, 3, 4, and 7 in cucumber revealed that only qMP2.1, qMP3, and qMP7 are associated with MPF traits. The number of nodes with MPF traits were detected in four QTL: qMP2.2, qMP3, qMP4, and qMP7. Notably, qMP2.2 on chromosome 2 had the most notable impact, with an LOD score of 7.58, closely followed by qMP3, on chromosome 3, with an LOD score of 7.53. This study highlights the effectiveness of QTL analysis regarding the number of female flowers per node, and the nodes with female flowers indicated that nearly all QTL were associated with the same SNP molecular markers or had closely adjacent positions. The only exception was qMP4 on chromosome 4, which exhibited a specific association with the trait related to the data on the number of nodes with more than one female flower. These QTL show promise for their potential use in supporting conventional breeding efforts to enhance efficiency and expedite selection and improvement processes, ultimately leading to higher cucumber yields and increased farmer’s income.

## Author Contribution Statement

Conceptualization, N.A., W.S., N.O., S.C. and J.K.; methodology, N.A., N.O., W.S., S.C. and J.K.; software, N.A.; validation, J.K., W.B. and N.A.; formal analysis, N.A.; N.O. and W.S.; investigation, J.K. and N.A.; resources, N.A., W.S., N.O. and S.C.; data curation, N.A., W.S., N.O. and S.C.; writing original draft preparation, N.A., W.S. and N.O.; writing review and editing, J.K. and W.B.; visualization, J.K., N.O. and N.A.; supervision, J.K., W.B., N.O. and N.A.; project administration, N.O., S.C. and N.A.; funding acquisition, N.O., W.S. and S.C. All authors have read and agreed to the published version of the manuscript.

## Figures and Tables

**Table 1.** Variety evaluation and homogeneity testing of six cucumber varieties

Variety	Phenotype description of flowering	Sex expression	Homogeneity (percentage)	Selecting evaluattion
EWSCU-809	MPF	Gynoecious	97.14^a^	Female line
EWSCU-173	MPF	Predominantly Gynoecious	95.95^ab^	Discard
EWSCU-735	MPF	Predominantly Gynoecious	94.76^bc^	Discard
EWSCU-536	MPF	Gynoecious	93.57^c^	Discard
EWSCU-989	SPF	Predominantly Gynoecious	95.24^abc^	Male line
EWSCU-076	SPF	Predominantly Gynoecious	90.24^d^	Discard
F-test			0.00	
CV (%)			1.19	

Means followed by the same letters indicate no differences at P ≤ 0.05 by the least significant. Multi-pistillate flowers (MPF). Single-pistillate flowers (SPF).

**Table 2.** Mean of number of pistillate flowers (PF) per node, number of nodes with multi-pistillate flowers (MPF) and the mid-parent heterosis

Accession/Heterosis	Phenotype of flowering	Generation	Average of PF per node	Num. of nodes with MPF
EWSCU-809 (P1)	Female line (MPF)	F_6_ (inbred line)	3.76 ± 0.54^a^*	19.50 ± 1.18^a^
EWSCU-989 (P2)	Male line (SPF)	F_8_ (inbred line)	0.46 ± 0.17^c^	0.00 ± 0.00^c^
EWSCU-991	F_1_ hybrid	F_1_	1.05 ± 0.07^b^	1.30 ± 1.16^b^
EWSCU-992	Segregating population	F_2_	Segregation	Segregation
Mean	—	—	1.76	6.77
F-test	—	—	0.00	0.00
CV (%)	—	—	18.63	16.44
Heterosis	—	—	–50.23%	–90.08%

*Means followed by the same letters indicate no differences at P ≤ 0.05 by the least significant.

**Table 3.** The segregation and chi-square (χ^2^) analysis of the F_2_ populations (219 plants) were carried out considering the average number of multi-pistillate flowers (MPF) per node and the number of nodes with MPF traits, categorized into non MPF, partial MPF, and strong MPF

Trait	Accession	Observed				Expected			χ^2^ (9:6:1)	P-value
		No. of non MPF (plant)	No. partially MPF (plant)	No. of strong MPF (plant)		No. of non MPF (plant)	No. partially MPF (plant)	No. of strong MPF (plant)		
The average no. of MPF /node	EWS-809 (P1)	0	0	10		0	0	10		
	EWS-989 (P2)	10	0	0		10	0	0		
	EWS-991 (F_1_)	10	0	0		10	0	0		
	EWS-992 (F_2_)	139	67	13		123.1875	82.125	13.6875	4.850	0.0885
The no. of nodes with MPF	EWS-809 (P1)	0	0	10		0	0	10		
	EWS-989 (P2)	10	0	0		10	0	0		
	EWS-991 (F_1_)	10	0	0		10	0	0		
	EWS-992 (F_2_)	113	85	21		123.1875	82.125	13.6875	4.850	0.0885

df = 2.0, α = 0.05, χ^2^ = 5.99.

**Table 4.** The statistically significant quantitative trait locus (QTL) scores were determined based on the average number of pistillate flowers (PF) per node and the presence average number of multi-pistillate flower traits identified in the F_2_ population

Trait*^a^*	QTL*^b^	Chr*^c^*	Flanking markers*^d^*	Start-end position (cM*)	Position (bp*)	LOD peak	Additive effect*^e^*	DOM*^f^*	PVE (%)*^g^*
APF	*qMP2.1*	2	CUCSNP-0009679–CUCSNP-0009699	87.525–92.598	19,036,261	4.23	0.132226	−0.06823	5.6
	*qMP3*	3	CUCSNP-0009679–CUCSNP-0009699	46.878–51.269	12,522,694	7.53	0.183583	−0.08286	10.3
	*qMP7*	7	CUGSNP-7-60–CUCSNP-0032394	49.914–52.922	13,452,982	4.53	0.152063	−0.00418	5.9
NNMPF	*qMP2.2*	2	CUGSNP-2-96–CUGSNP-2-95	83.258–86.717	8,238,869	7.58	2.05504	−0.10091	10.6
	*qMP3*	3	CUCSNP-0011238–CUCSNP-0011410	46.878–51.269	12,522,694	4.99	1.79866	−0.10814	6.8
	*qMP4*	4	CUCSNP-0017116–CUCSNP-0017117	6.892–14.892	1,814,861	4.29	1.59174	−0.52615	5.8
	*qMP7*	7	CUGSNP-7-60–CUCSNP-0032394	49.914–52.922	13,452,982	6.9	1.95556	0.117892	9.6

* cM: Centi Morgan, QTL: quantitative trait locus, bp: base pair. *^a^* APF: Average number of pistillate flower/node, NNMPF: Number of nodes with MPF. *^b^* QTL: Designation of the QTL is in accordance with the rules recommended by McCouch (2008). *^c^* Chr: Chromosome. *^d^* LOD peak: Logarithm of the odds ratio (LOD) score of ≥2.5 was set as threshold for this data. *^e^* Additive effect: The value indicates the decrement in the trait value. *^f^* Dom: Dominance effect. *^g^* PVE (%): Total phenotypic variance (PVE%) in percentage explained by the QTL.
